# Formal analyses are fundamental for the definition of honey, a product representing specific territories and their changes: the case of North Tyrrhenian dunes (Italy)

**DOI:** 10.1038/s41598-023-44769-1

**Published:** 2023-10-16

**Authors:** Valeria Leoni, Sara Panseri, Luca Giupponi, Radmila Pavlovic, Carla Gianoncelli, Stefano Sala, Valeria Zeni, Giovanni Benelli, Annamaria Giorgi

**Affiliations:** 1 Department of Agricultural and Environmental Sciences - Production, Landscape, Agroenergy (DISAA), University of Milan, Via Celoria 2, 20133 Milan, Italy; 2 Centre of Applied Studies for the Sustainable Management and Protection of Mountain Areas (CRC Ge.S.Di.Mont.), University of Milan, Via Morino 8, 25048 Edolo, BS Italy; 3 Department of Veterinary Medicine and Animal Sciences (DIVAS), University of Milan, Via Dell’Università, 6, 26900 Lodi, Italy; 4 Fondazione Fojanini Di Studi Superiori, Via Valeriana 32, 23100 Sondrio, Italy; 5 Department of Agriculture, Food and Environment, University of Pisa, Via del Borghetto 80, 56124 Pisa, Italy

**Keywords:** Plant sciences, Environmental sciences

## Abstract

Honey is a variegate matrix depending significantly on the floral origin, and it could become an important agri-food product to valorise specific territories. Being so diverse, different analytical techniques are necessary for its description. Herein we characterized the honey produced in one of the Italian sand dunes systems hosting beekeeping activities. In terms of floristic origin, phytochemical characterization, and sensory and colour analysis, honey collected in 2021 and 2022 was comparable. Honey was polyfloral, with several pollens from dune habitat plants classified as minor. The presence of the allochthonous *Amorpha fruticosa* L. and the ruderal *Rubus fruticosus* L. pollens in the category of the secondary pollens testifies the alteration of the park vegetation. The phytochemical profile was rich in polyphenols. Other interesting compounds were coumarine derivatives, likely attributable to resin-laden plants as rockroses, long chain hydroxyacids typical of royal jelly and nicotinic acid and its analogues (2-hydroxynicotinic acid and 2-hydroxyquinoline). The above-mentioned honey showed interesting features and was a good representation of the vegetation of this area. Our study pointed out the importance of relying on multiple analytical techniques for the characterization of honey and the advisability of a technical support toward beekeepers to correctly describe and valorise their product.

## Introduction

Honey is the sweet substance produced by *Apis mellifera* L. (Hymenoptera: Apidae) from nectar and, less often, from the excretions of some phytophagous insects or other sweet plant excretions. This substance is then converted by digestive enzymes of honeybees and stored and dried in the honeycomb, where the nectar totally turns into honey^1^.

The main distinction among honeys is due to their floristic origin and they can be classified as monofloral (or else unifloral) or multifloral/polyfloral^1^. The first is a product with a principal floristic origin, whilst the latter is the outcome of a specific blend of nectars, none of which is predominant in the defining of honey organoleptic properties. Honey is a complex matrix, composed by macronutrients as carbohydrates (mainly fructose and glucose) and other minor substances, among which secondary metabolites of plants^2,3^. It presents great variation in composition and features depending on the floral origin^2,4^.

Since the features of honey are very much linked to the flower nectar foraged by bees, it is easy to understand how different environments with a different vegetation can originate specific kinds of honey. Although difficult in practice, to standardise specific types of honey is important for beekeepers, and honey can become an important product to valorise specific habitats and territories, as in the case of wildflower mountain honey^5^, becoming the description of those habitats, their vegetation and, besides, the environmental and social (as abandonment or changes in use) changes happening to them. There are many other cases where honey from a specific territory was characterised. Belay et al.^6^ described the physicochemical properties of the Harenna forest honey, in Ethiopia, while Stagos et al.^7^ investigated different types of honey derived from Mount Olympus in Greece. Otmani et al.^8^ described bitter and sweet honey of Algeria Mediterranean coast. Sanz et al.^9^ characterized artisanal honeys produced by small-scale apiculturists in the mountain area of Madrid.

However, a territorial definition solely could not be used on the label of honey supplies. The floristic source is the only classification envisaged by the European legislation. This information is not considered obligatory to specify in the label, although^10^. The floristic source is whether blossom or plant a specific honey predominantly comes from, associated with the appropriate organoleptic, physicochemical, and microscopic properties corresponding to that origin^1,10^. What derives from this legislative framework yet is that very often the classification of honey is commercially performed without carrying out melissopalynological analysis. The botanical origin of honey is often based on the claims of local beekeepers, or by considering the predominant flowers surrounding the hive, or even considering very subjective taste and smell evaluations. The official analysis to define the floristic origin of honey is indeed the melissopalynological analysis, i.e., the study of the pollen residues in honey^11^.

Basing on melissopalynology, honey is defined “unifloral” when a specific plant pollen accounts for more than 45% of all pollens, while in other circumstances a little amount is sufficient. This is applicable for honeys obtained from plants that either yield little pollen or produce pollen that is under‐represented in honey, like *Robinia pseudoacacia* L. honey^12,13^. Other than melissopalynology, other criteria are important to define the floristic origin of honey, as sensory analysis by expert tasters and physical–chemical analysis.

In fact, melissopalynological analysis alone may not adequately describe honey. Weak pollen producers are very common among melliferous plants (and many of them have not been studied yet in this regard) or plants are visited by honeybees for other purposes apart from foraging nectar. In these cases, phytochemical profiling is a useful instrument to provide additional information to melissopalynology and tasting. For example, flavonoid profile appears to be linked with the nectar sources^14,15^ as well as volatiles substances^16,17^ that define the aroma of the product. Aroma acts in synergy with other factors such as taste and physical aspects (e.g., colour and consistence) in defining the flavour of honey^18^. Therefore, plant-derived compounds and their metabolites (terpenes, norisoprenoids and benzene compounds and their derivatives) can become effective markers of the floral origin of honey^19^.

Italy, due to its geomorphological features, hosts many different habitats with their variegate flora, and honey is then an important agri-food product^20^, useful to describe and valorise this richness. Shores represent an important and widespread habitat. For example, coastal protected areas in Tuscany, constituted by sandy beaches and dunes, are territories of naturalistic, touristic, and economic importance, and host fauna and flora especially adapted to this habitat. Migliarino-San Rossore-Massaciuccoli Regional Park is a protected area hosting one of the largest Italian sand dunes systems, where also few beekeeping activities are carried out. The exceptional vegetation of these habitats could give special features to honey produced in this part of the Tyrrhenian coast, that is also included in Tuscany Agro-alimentary Traditional Products (PAT) list.

Regardless the interest of stakeholders and the importance of the flora of the dune system of this Regional Park, this honey is still poorly characterized and described without the background of a deep investigation. The aim of this work was then the characterization of honey produced in Migliarino-San Rossore-Massaciuccoli Regional Park through botanical origin (melyssopalynological analysis), sensory analysis and the definition of colour/colour intensity. The phytochemical composition was investigated through chromatographic techniques (Solid Phase Micro Extraction-Gas Chromatography/Mass Spectrometry, SPME–GC–MS, and High-Performance Liquid Chromatography/Mass Spectrometry Orbitrap, HPLC-Orbitrap). The features of the honey produced in the above-mentioned Regional Park were studied on samples produced over two years.

## Material and methods

### Sampling area

Migliarino-San Rossore-Massaciuccoli Park (Latitude: 43°43′28″; Longitude: 10°20′17″) is a protected natural area established by LR Toscana n. 61 of 13 December 1979 (L.R. 13 December 1979, n. 61). According to the classification of the ecoregions of Italy^21^ this area is within the Northern and central Tyrrhenian Section (Tyrrhenian Province, Mediterranean Division) and has annual medium temperatures above 13 °C and an all-year growing season, with annual precipitation up to 1400 mm and a summer decrease, which determines more than 2- or 3-month-long aridity.

One of the remarkable habitats hosted by Migliarino-San Rossore-Massaciuccoli Park are sand dunes. Dunes are coastal landforms made up of sand sediments^22^. One of the most important actors for their development is vegetation, that stops the advancement towards inshore of the sediments moved by the wind^23–25^. This differentiates them from continental mobile dunes. What derive is a complete dunal system made of beach (totally lacking vegetation), the active foredunes, the interdunes and the stable dunes^25–27^. The main plant association that occupies the foredune in the part nearest to the sea is *Salsolo kali-Cakiletum maritimae* (constituted by annual halonitrophilous plants, such as *Salsola kali* L. or *Polygonum maritimum* L., able to stand at the strongest stresses due to wind, salt and sand movement. *Echinophoro spinosae-Elymetum* association occupies the mobile foredunes, while on the remotest part of the mobile dunes we can find the *Echinophoro spinosae-Ammophiletum arundinaceae* association, with species as *Ammophila arenaria* (L.), *Eryngium maritimum* L., *Euphorbia paralias* L. and *Pancratium maritimum* L.^28,29^. The interdune is already made of stabilized dunes characterized by the *Pycnocomo rutifolii-Seseletum tortuosi* association. This type of habitat is dominated by *Helycrisum* spp., *Seseli tortuosum* L., *Pancratium maritimum*, and *Solidago litoralis* Savi in the damper areas^29^. In the interdunal belt is remarkable also the *Vulpietum fasciculatae-Silenetum coloratae* association^30^ with the presence for example of *Ononis variegata* L., *Pseudorlaya pumila* (L.) Grande, *Cutandia maritima* (L.) Barbey, *Medicago littoralis* Loisel., *Lagurus ovatus* L.^29^. In the backdune the softer action of the wind and the presence of organic matter in the soil allows the occurrence of shrubs forming the *Asparago acutifolii-Juniperetum macrocarpae* association, with medium tall shrubs such as *Juniperus oxycedrus* L. subsp. *macrocarpa* Sibth. & Sm., *Pistacia lentiscus* L., and *Genista* spp.^29^. In this typically Mediterranean scenario, we can find *Cistus* spp., with anthesis from April to June, and *Olea europea* L. var. *sylvestris.* Passing progressively to a more continental ecosystem^28^ we find the presence of tall shrubs and trees as *Myrtus communis* L., *Ligustrum vulgare* L. or *Rhamnus alaternus* L, that finally mix with tall trees as *Pinus pinaster* Aiton and *Pinus pinea* L. that are residues of a reforestation, or the natural potential of the Tyrrhenian Province, that is *Quercus ilex* together with mixed *Q. suber* forests (*Quercetea ilicis* phytosociological class).

In Migliarino-San Rossore-Massaciuccoli Park, however, are comprised also the wet areas surrounding Massaciuccoli lake. This area of naturalistic importance hosts the biggest coverage of *Cladium mariscus* L. in Italy, together with other marsh plants as *Lemna minor*, *Ceratophyllum demersum*, *Lemna gibba*, *Osmunda regalis*, *Typha latifolia* and *Typha angustifolia* and the more common *Carex* spp. and *Juncus* spp. The naturalistic value of Lake Massaciuccoli has significantly decreased in recent years due to eutrophication of water, an unresolved problem that caused the alteration of natural vegetation^31^ and the occurrence of allochthonous plants. Nearby, we find the large urban centres of the city of Pisa and Viareggio, in the province of Lucca, and the related transport infrastructures.

The park grants, under an agreement, the right to some agricultural holdings to raise honeybees. The honey samples studied here were collected in 2021 (3 pots, samples A) and 2022 (3 pots, samples B), and analysed in 2022.

### Melissopalynological analysis

Melissopalynological analysis were performed according to the procedures recommended by the International Commission for Bee Botany (ICBB) for qualitative melissopalynological analysis^32^.

To prepare the slides, 12 g of honey were dissolved with 40 mL of distilled water in a 50 mL test tube with conical bottom. The solution was centrifuged for 15 min at 3000 rpm, then the liquid was separated from the sediment by aspiration. Then the sediments were dissolved again in 10 mL of distilled water and centrifuged again for 5 min at 3000 rpm, the supernatant was eliminated by pouring gently. The sediment was carefully dispersed with a Pasteur pipette on a glass slide on an area of about 1 cm^2^. After the sediment dried up, it was included in a drop of glycerol gelatin, previously dissolved in a water bath, and covered with a coverslip.

To provide then the percentage of specific pollens, at least 300 pollen grains were counted for an estimation of the relative frequencies of pollen types^33^. A count of abortive, irregular, or broken pollen grains, fungal spores, hyphae, and microscopic algae, if they could be identified, was performed as well. The pollen types present in the honey samples were identified, counted, and classified, according to their percentages, as dominant pollen (more than 45% of the total pollen grains counted), secondary pollen (from 16 to 45%), important minor pollen (from 3 to 15%), minor pollen (less than 3%) or sporadic pollen (less than 1%)^34^.

### Phytochemical analysis

The phenolic compounds were extracted by sugaring-out assisted liquid–liquid extraction (SULLE) in the modified LLE version for the analysis of honey^35–37^ using a sodium chloride solution instead of deionized water for dissolving honey for better phase separation and increased recoveries of polyphenols.

In details 0.5 g of honey sample were placed in a 2 mL round bottom Eppendorf polypropylene tube and 10% NaCl solution (0.5 mL) in 0.01 M HCl (pH 2) was added. The tube was then agitated in a vertical tube vortex mixer until all the solids were dissolved. MeCN (1 mL) was added to the mixture and the tube was vortexed for another 1 min at 2000 rpm, followed by centrifugation for 1 min at 15,000 rpm. The upper organic phase was collected in a 2 mL crimp top chromatography vial. Another portion of MeCN (1 mL) was added and the extraction procedure was repeated one time, with the total collected organic phase amount of about 1.9 mL. The organic phase was dried under a gentle nitrogen flow at room temperature and reconstituted in 98:2 v/v water/MeCN mixture (0.5 mL) with 0.1% FA added. The extracts were stored at 4 ◦C in the dark prior to the analysis. A QC sample was prepared and analysed in the same batch^38^.

The extract was filtered by 0,45 um syringe filter and subsequently diluted with 0.1% of formic acid (1:1). An amount of 10 μL was introduced in HPLC–Q-Exactive-Orbitrap®–HRMS instrumentation. Untargeted metabolomics approach was performed according to the recently developed strategy for propolis^39^ and mountain wildflower honey^5^. All analysis were repeated for three biological samples.

The volatiles profile was performed through HS–SPME–GC–MS analysis of Volatiles Organic Compounds (VOCs). All the samples were prepared by weighing exactly 5 g of honey in a 20 mL glass vial along with 10 μL of the IS (4-Methyl-2-pentanone, 2 mg/L in 2-propanol). Each vial was fitted with a cap equipped with a silicon/PTFE septum (Supelco, Bellefonte, PA, USA) and passed in an ultrasonic bath for 10 s at 30 °C. At the end of the sample equilibration period (1 h), a conditioned (1.5 h at 280 °C) 50/30 μm Divinylbenzene/Carboxen/polydimethylsiloxane (CAR/PDMS/ DVB) StableFlex fibre (Supelco, Bellefonte, PA) was exposed to the headspace of the sample for the extraction (120 min) by CombiPAL system injector autosampler (CTC analytics, Switzerland). The fibre and the time of extraction used in this study were selected after preliminary studies. The extraction temperature of 25 °C was selected to prevent possible matrix alterations (oxidation of some compounds, particularly aldehydes and furans). To keep a constant temperature during analysis, the vials were maintained on a heater plate (CTC Analytics, Zwingen, Switzerland).

GC–MS analysis was performed using a Trace GC Ultra (Thermo-Fisher Scientific, Waltham, MA, USA) Gas Chromatograph coupled to a Trace DSQII quadrupole mass spectrometer (MS) (Thermo-Fisher Scientific, Waltham, MA, USA) and equipped with an Rtx-Wax column (30 m; 0.25 mm i.d.; 0.25 μm film thickness, Restek, USA). The oven temperature program was from 35 °C, hold 8 min, to 60 °C at 4 °C/min, then from 60 to 160 °C at 6 °C/min, and finally from 160 to 200 °C at 20 °C/min, hold 20 min. Carryover and peaks originating from the fibre were regularly assessed by running blank samples. After each analysis, fibres were immediately thermally desorbed in the GC injector for 5 min at 250 °C to prevent contamination. The injections were performed in splitless mode (8 min). The carrier gas was helium at a constant flow of 1 mL/min. The transfer line to the mass spectrometer was maintained at 230 °C, and the ion source temperature was set at 250 °C. The mass spectra were obtained by using a mass selective detector with the electronic impact at 70 eV, a multiplier voltage of 1456 V, and by collecting the data at rate of 1 scan s^−1^ over the m/z range of 35–350. Compounds were identified by comparing the retention time (RT) with the literature data and through the National Institute of Standards and Technology (NIST) MS spectral database as in previous research^40,41^. Volatile compounds measurements from each headspace of honey extracts were carried out by peak area normalization (expressed in ppm).

All analyses were repeated for three biological samples. In Tables S1 and S2 the content of each compound revealed in the HPLC–Q-Exactive-Orbitrap®–HRMS and (SPME) GC–MS analyses respectively is reported as mean ± SD of the quantification performed on each biological sample. Once the assumptions of normality of group data and homogeneity of variances were verified, p-values were calculated through an unpaired t-test to verify if the samples contained the compounds in a significantly different quantity (indicated by a code of statistical significance in Tables S1 and S2). Heatmap was performed on the compounds resulted significantly different to highlight the differences among honey samples. The heatmap and hierarchical cluster analysis and the other statistical analysis were performed using R 3.5.2. software^42^, gplots package, heatmap.2.

### Colour analysis and sensory description

To determine the colour, solutions of honey with distilled water at 50/50 w/v % concentration were heated to 50° until sugars were completely dissolved. Absorbance was measured at 635 nm, and absorbance values were classified according to the Pfund scale^43,44^ with the formula:

$$ {\text{Pfund}}\;{\text{index}}\;\left( {{\text{mm}}} \right) = - {38}.{7}0 + {371}.{39}*{\text{absorbance }}\;\lambda {635} $$

Colour intensity can be defined instead as the net absorbance between two absorbance values (nm), corresponding to the difference of absorbance (expressed as mAU)^45^:

$$ {\text{Colour}}\;{\text{intensity}}\;\left( {{\text{mAU}}} \right) = {\text{absorbance}}\;\lambda {45}0 - {\text{absorbance}}\;\lambda {72}0 $$

During the spectral acquisition distilled water was used as blank.

The analyses were performed at room temperature (25 °C) and confronted with known clear—medium—dark and more or less intense coloured honey (light and clear: rhododendron; medium and medium intense: hedysarum; medium and intense: chestnut; dark and intense: manuka and honeydew; dark and very intense: heater). For each kind of honey two biological samples were considered for the spectrophotometric analysis and values are showed in Fig. 3.

A professional taster described the honey through sensory analysis. Odour and aroma were described following the odour and aroma wheel as described in^46^.

## Results

The melissopalynological analysis showed that honey samples from Migliarino-San Rossore-Massaciuccoli Regional Park were “polyfloral” with no dominant pollen and secondary pollens belonging to *Amorpha* and *Rubus* genera, in different percentages depending on the years (Table 1). Pollens typical of the dune habitats were present in the category of minor pollens or even sporadic pollens such as *Umbelliferae*, *Compositae*, *Ononis, Cistus, Helianthemum* for herbaceous species, and *Genista* spp. and *Myrtus* f. considering shrubs. More than half of the pollen types (58%) were present both years, all the secondary and minor pollens were identical, and some qualitative differences were found only in the sporadic pollens category (Table 1).

**Table 1:** Relative frequencies of the main pollen types (in percentage) retrieved in the two honey samples analyzed (2021 and 2022).

		2021	2022
Dominant pollen (> 45%)		–	–
Secondary pollen (16–45%)	*Rubus*	38.60	26.37
	*Amorpha*	30.03	20.51
Minor pollen (2–15%)	*Sedum*	4.95	4.39
	*Lotus corniculatus*	4.60	3.12
	*Trifolium repens gr*	3.96	6.59
	*Cruciferae (Sinapis f.)*	3.63	4.02
	*Genista*	2.64	6.22
	*Clematis*	1.65	3.29
	*Trifolium pratense* gr	1.32	3.29
	*Umbelliferae*	–	6.95
	*Hedysarum*	–	6.22
	*Compositae H*	1.32	–
	*Astragalus/Ononis*	–	2.19
Sporadic pollen (1% or less)	Umbelliferae	≤ 1	–
	*Hedysarum*	≤ 1	–
	*Compositae H*	–	0.73
	*Astragalus/Ononis*	≤ 1	–
	*Pyrus/Malus*	≤ 1	≤ 1
	*Myrtus f*	≤ 1	≤ 1
	*Cornus sanguinea*	≤ 1	≤ 1
	*Ailanthus*	≤ 1	≤ 1
	*Aster/Solidago*	≤ 1	≤ 1
	*Galega f*	≤ 1	≤ 1
	*Rhamnus*	≤ 1	≤ 1
	*Helianthemum*	≤ 1	≤ 1
	*Acer*	≤ 1	≤ 1
	*Diplotaxis f*	≤ 1	≤ 1
	*Quercus r*	≤ 1	≤ 1
	*Quercus ilex*	≤ 1	≤ 1
	*Fraxinus/Olea*	≤ 1	≤ 1
	*Sambucus*	≤ 1	≤ 1
		≤ 1	≤ 1
	*Gleditsia*	≤ 1	–
	*Prunus*	≤ 1	–
	*Centaurea j*	≤ 1	–
	*Ligustrum*	≤ 1	–
	*Hedera*	≤ 1	–
	*Salix*	≤ 1	–
	*Citrus*	≤ 1	–
	*Ranuncolaceae*	≤ 1	–
	*Euphorbia*	≤ 1	–
	*Potentilla*	≤ 1	–
	*Trifolium incarnatum*	≤ 1	–
	*CistusPapaver*	≤ 1	–
	*Mercurialis*	≤ 1	–
	*Carex*	≤ 1	–
	*Chelidonium*	≤ 1	–
	*Boraginaceae(Cerinthe*.*Cynoglossum f.)*		
	*Tamarix*	–	≤ 1
	*Parthenocissus*	–	≤ 1
	*Medicago*	–	≤ 1
	*Castanea*	–	≤ 1
	*Carex*	–	≤ 1
	Graminaceae	–	≤ 1

**Figure 1:**
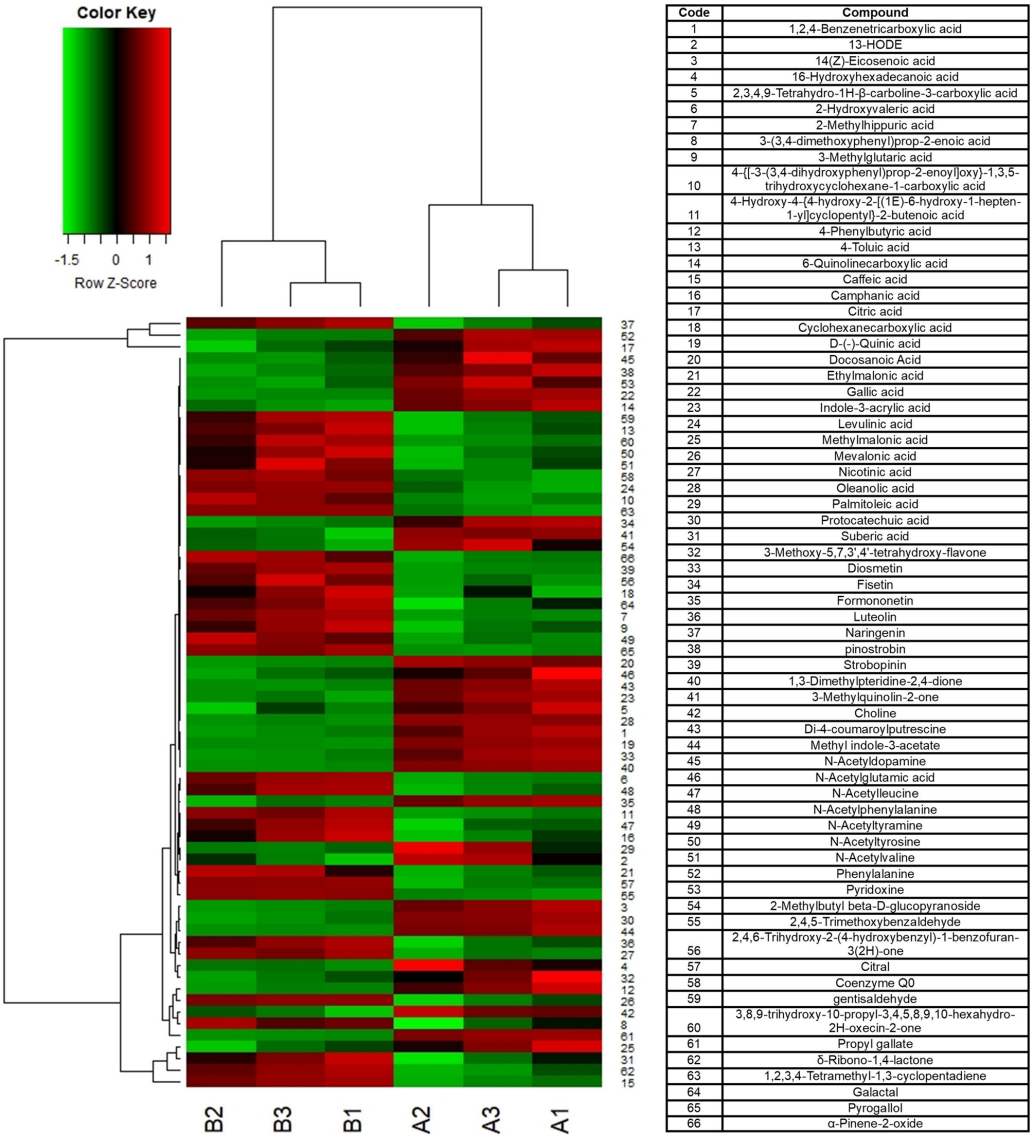
Hierarchical cluster analysis: heat-map reflecting the differences between compounds revealed in HPLC-Q-Exactive-Orbitrap-MS metabolomic analysis of secondary metabolites. Only the compounds significantly different in the t-test (Table S1) are considered. Capital letters indicates the sample (**A** 2021; **B** 2022) and the number the biological repetition.

Honey produced in this area resulted very rich in polyphenols (Table S1). Flavonoids found included daidzein, apigenin, chrysin, naringenin, kaempferol, genistein, pinostrobin and quercetin. Caffeic acid was another noteworthy phenolic compound present in a significant amount (68.61 ± 1.26 ppm in 2021and 80.72 ± 1.09 ppm in 2022). Naringenin was also one of the main compounds, found in a quantity of more than 100 ppm both years (160.56 ± 6.87 ppm in 2021 and 190.61 ± 4.39 ppm in 2022), together with 4-Hydroxycoumarine, 7-Hydroxycoumarine, Citric acid, and Benzophenone. Lactic acid was present in a good amount (195.23 ± 30.70 ppm and 180.29 ± 1.78 ppm). Other interesting compounds were acetylated and not-acetylated amino-acids (e.g., phenylalanine was found in an interesting amount, 175.27 ± 6.38 ppm and 138.35 ± 2.99 ppm respectively in 2021 and 2022; other important amino-acids were valine, leucine, and isoleucine) and some fatty acids as palmitic and suberic acid. Finally, few long chain hydroxy-acids were found (i.e. 9-HODE, 10-HDA and 13-HODE). Some other interesting metabolites were nicotinic acid and its analogues (2-Hydroxynicotinic acid and 2-Hydroxyquinoline). The secondary metabolites profile was qualitatively similar the two years (Table S1), as happened for pollens, with some significant quantitative differences for 46 compounds out of 140 (Fig. 1).

**Figure 2:**
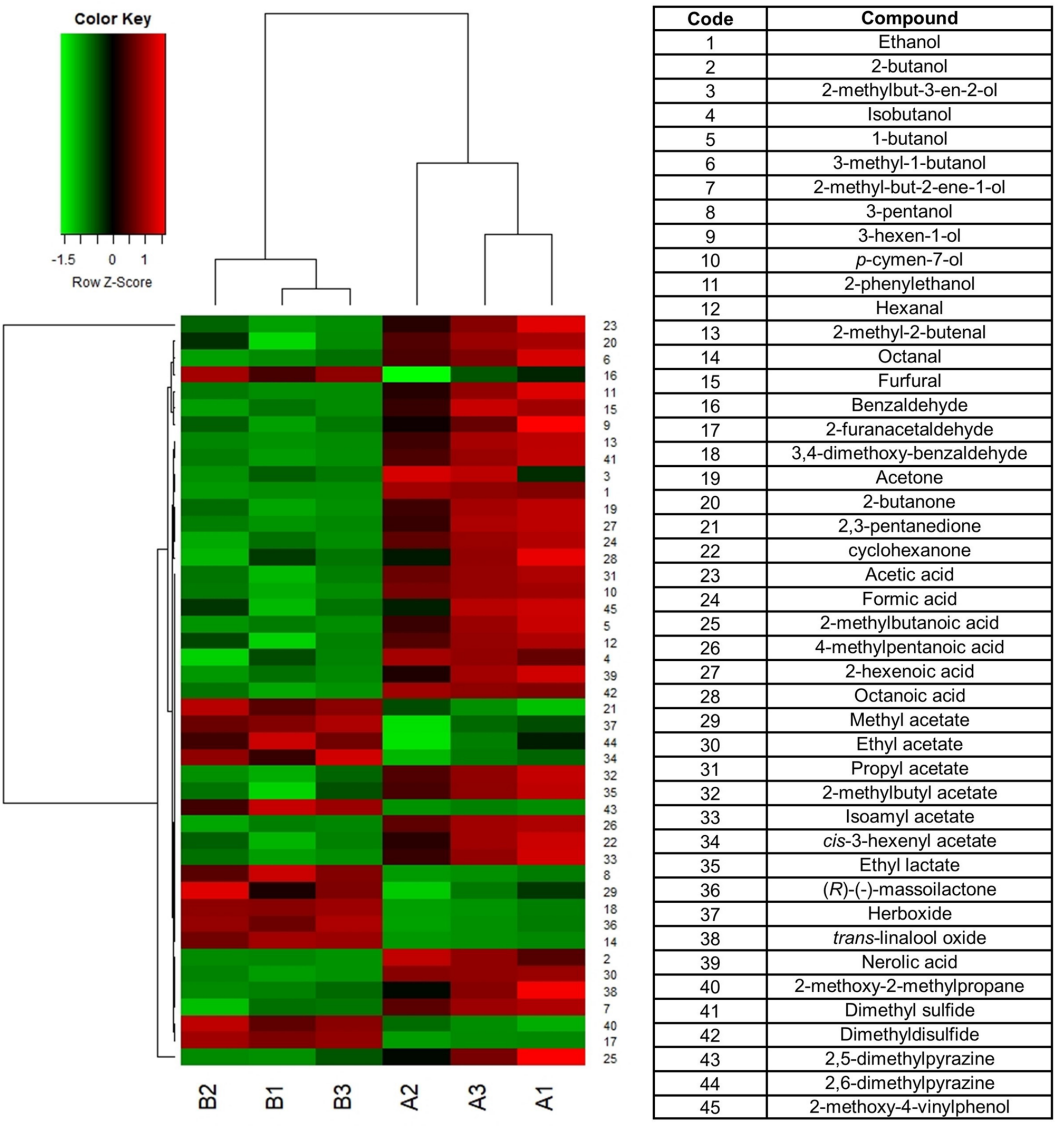
Hierarchical cluster analysis: heat-map reflecting the differences between compounds revealed in the (SPME) GC–MS analysis of volatiles compounds. Only the compounds significantly different in the t-test (Table S2) are considered. Capital letters indicates the sample (**A** 2021; **B** 2022) and the number the analytical repetition.

The volatiles profile as well was qualitative similar and quantitatively different for about half of the compounds (Table S2; Fig. 2), with a somewhat marked difference between 2021 and 2022, mainly due to some compound as acetic acid (333.11 ± 14.10 ppm in 2021 and 292.41 ± 4.58 in 2022). Other important compounds were some carboxylic acids as propanoic acid, isobutyric acid, butyric acid and 2-methylbutyric acid.

**Figure 3:**
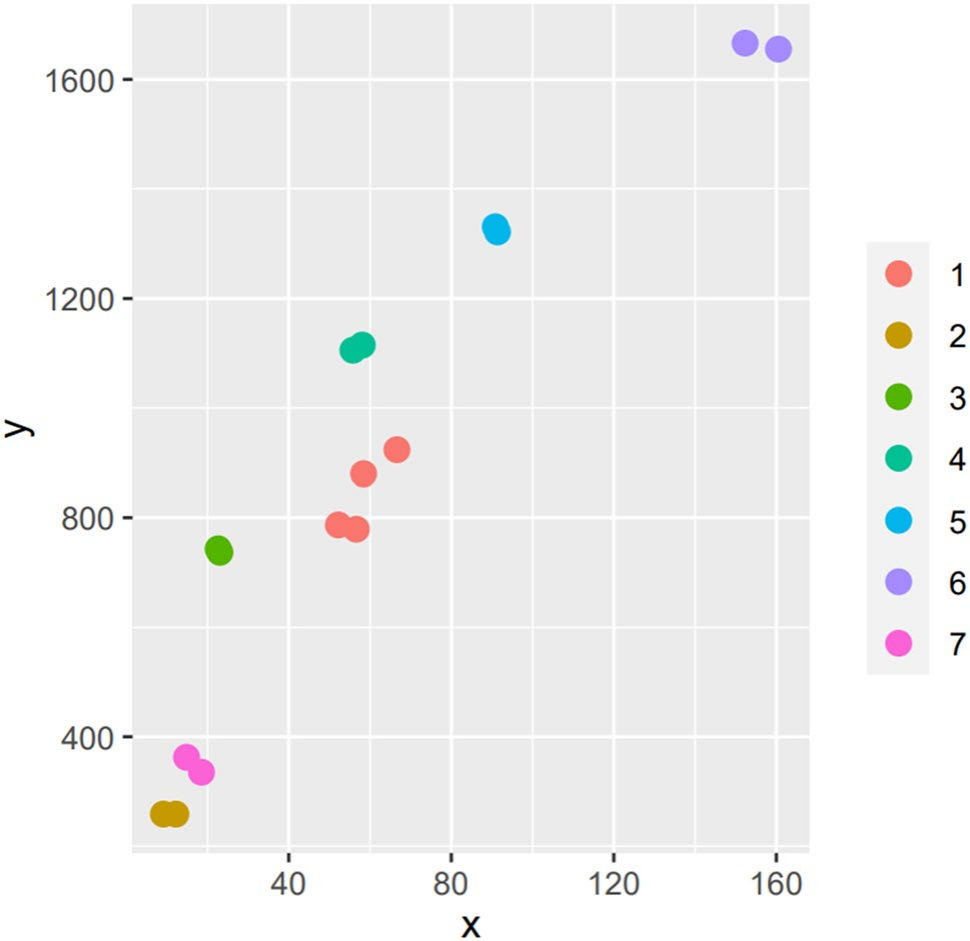
Colour of the honey analysed compared with other known honey (axes y colour; x colour intensity). Code samples: Honey from Migliarino-San Rossore—Massaciuccoli park (1); Rhododendron (2); Chestnut (3); Honeydew (4); Manuka (5); Heather (6); Hedysarum (7).

According to Pfund scale^43,44^ and colour intensity^45^, the honey analysed resulted medium dark (near honeydew and chestnut), deep in colour (Fig. 3), deeper than *Hedysarum* honey but less than heather/calluna, a known honey with intense colour^46^. Both sampling years honey was described as warm, caramelised, and cooked fruit-like, almost burned. Notes of dried fruits were evidenced (e.g., candied nuts, sultanas, figs) and the 2021 sample had also a fresher/fruity note not shown in the 2022 sample. The aroma was described as fatty and warm, strong, acidic with citrus and herbal tea notes.

## Discussion

The results of the analyses permitted a deep and multidisciplinary investigation on the honey produced in such a specific territory. For beekeepers and small-medium producers, honey produced in exceptional ecosystems has always carried a significant territorial connotation^5–9^. However, the classification and description of honey frequently rely on common knowledge without a formal analysis and claims that these honeys have health properties are frequently made without appropriate scientific evaluations^47^.

In Italy, the entire coastline is about 7500 km, and about a half of it is represented by sandy beach-dune systems. Of these, few parts are well-maintained and present the aspect and the vegetation described previously due to disturbing factors as urbanization, industrialization and the connected transport infrastructures, tourism, farming practices, erosion, pollution, and invasion from alien species^25,48–51^.

In our case study, an important contribution to honey was given by *A. fruticosa* (false indigo; Table 1), a deciduous shrub native to eastern and south-eastern North America and introduced to Europe at the beginning of the eighteenth century as an ornamental plant^52^. This plant is considered an invasive species (https://gd.eppo.int/taxon/AMHFR/distribution), and inhabits especially humid areas where it grows on loamy or sandy soils with low nutrient contents^52^, altering the local ecosystem^53^. This plant has a high melliferous potential, and it is now appearing more and more frequently as unifloral honey^16^, as happened for other plants invasive in Italy as *R. pseudoacacia* or *Ailanthus altissima* (Mill.) Swingle. The occurrence of allochthonous flora and the alteration of expected vegetation is well reported in Migliarino-San Rossore-Massaciuccoli Park^31^, and the degraded industrial and urban surrounding the park are numerous. Another important essence detected in the melissopalynological analysis was *Rubus* (Table 1), nay *R. fruticosus* L. (melissopalynological analysis does not distinguish the main species of *Rubus*, *R*. *fruticosus* and *R. idaeus*, which are instead inferred by the production area). The *Rubus* genus comprises many invasive and non-invasive species^54^ and *R. fruticosus* is a shrub typical of very disturbed areas, as railways and industrial areas. Nevertheless, we could also find groups of plants characteristic of the dunal system, as *Compositae* H (between them *Helycrisum* spp.), *Euphorbiaceae* family, *Solidago* and *Ononis* genus, *Cistaceae* among which *Helianthemum* spp. and species of the back dunal system as *Genista* spp. and *Myrtus communis.* Among the not nectariferous we found also typical tree species of the Mediterranean garrigue as *Olea europea* var. *sylvestris*, *Quercus suber* and *Q. robur.* Finally, we found *Pinaceae* pollens, coherently with the area description.

The botanical origin has been already described as correlated with the profile of secondary metabolites as polyphenols or volatiles compounds^14,15^ and the search of markers is seen as one of the modern methods for assessing the quality of honey and its botanical origin identification^55–58^. A significant amount of naringenin was found in other varieties of honeys^59^, and the honey analysed was found particularly rich in phenolic compounds, in variety (more than twenty different flavonoids were identified) and quantity (Table S1). Other than being important for the organoleptic properties of honey, phenolic compounds seem to have a role also in its health properties^60–62^. In this regard, the presence of few long chain hydroxyacids was interesting (i.e., 9-HODE, 10-HDA and 13-HODE). These compounds are characteristic of royal jelly and are also found sometimes in some kinds of honey^63^, so they could be responsible of some health properties of honey^64–66^ since royal jelly is a well know nutraceutical bee product.

The presence of coumarin derivatives (in our case 4-hydroxycoumarine and 7-Hydroxycoumarine, Table S1) was as well notable and this group of molecules was already found in propolis^67^. Propolis, or bee glue, is a natural wax-like resinous substance that bees use to seal open spaces in the beehive and to prevent contamination inside the hive by bacteria, viruses, or parasites because of its antiseptic effect^68^. In the species distinctive of the flora of the area and honey samples considered, we could find different resin-laden plants, ad *Myrtus communis* or, especially, rockroses (*Cistaceae*), perennial herbaceous plants growing in open areas of infertile soils indigenous to the Mediterranean region^30^. Most *Cistus* species have aromatic foliage, and some species are known to exude a highly aromatic gums and resins^69^. 7-hydroxycoumarin is commonly called Umbelliferone, and very likely is present also in different *Apiaceae* species of the dunal ecosystem, as could be *Crithmum maritimum* L. or *Echinophora spinosa* L., and rockroses have been already described as responsible of the special sensorial properties of honey coming from the Mediterranean garrigue, for example Sardinian honey (Carla Gianoncelli, personal communication). In this case is possible to see how a multi-technique approach is important to give cues on the description of a complex matrix as honey, that in this case is considered a traditional product of the area.

The Honey produced in Migliarino-San Rossore-Massaciuccoli Regional Park is in fact included in the Tuscan PAT list with the D.L. n°173/98, Art. 8 (decree of the Italian Ministry of Agricultural, Food and Forestry Policies- MiPAF number 350/99) as “Miele di Spiaggia” (“foreshore honey”). It is to mention that this territorial definition is just supplementary to the one of “polyfloral honey”, clearly indicated on the label of this honey. In the description both in the labels and the regional decree, moreover, are mentioned some species considered responsible of the especial scent and organoleptic properties of this honey, as *Helichrysum* spp. (*H. stoechas* for example, or *H. italicum*), together with rockroses and *Arbutus unedo* L., and allochthonous as *Pittosporum tobira* (Thunb.) W.T. Aiton and *Tamarix* spp. However, we could not find some of the pollens belonging to the plants mentioned in the decree. Especially *A. unedo* is a very characterizing nectar source, and its presence gives a strongly bitter taste to honey (C. Gianoncelli, personal communication). This was not verified in our case, also if honey was produced in the PAT area and the slightly bitter aftertaste is indicated in the technical sheet of this honey.

In the decree is also described how the floral origin of the honey is limited to the Mediterranean garrigue due to “the complete isolation of this coastal strip due to the pine forest that, for insects, is an impenetrable limit”. This is partially a discrepancy with our findings, since a great part of pollen was from *A. fruticosa*, very likely found by bees in the humid areas around Massaciuccoli lake, and *R. fruticosus*, a ruderal plant of very disturbed peri-urban areas. Nevertheless, this is not in contrast with the assertion that this honey is the representation of the park environment, since we mentioned may times that many areas in the park are subjected to a strong habitat degradation. Honeybees forage across areas covering several hundred square kilometres, and at linear distances as far as 9 km from the hive^70^. Under extraordinary conditions, such as low availability of resources or a particularly rich flower patch, they can cover a foraging distance up to 12 km^71^. The vegetation of the dunes system is not composed mainly by nectar producer species and the habitat can be considered as a poor foraging territory for bees, subjected to environmental stresses as wind and salt. So, in some condition of draught or stress, or environmental changes, the rear pine forest could no longer represent an “impassable” limit, as mentioned in the decree that besides dates to more than twenty years ago. In a complex matrix as honey that is affected by such changes to fix precise features and properties is difficult.

For long time, honey produced in the littoral Northern Tuscany area was even called “helichrysum honey” giving an “impossible” floral origin, since some *Helichrysum* spp. have been described as non-nectariferous plant^72^, although they were also described as strongly entomophilous^73^. There are some studies reporting that *A. mellifera* forages on *Helichrysum* species^74^ although, as many other species of *Compositae* family, pollinators of many different orders are reported to visit this plant genus. More studies should be performed on the pollination ecology of the plants composing this sand dunes system. It is unclear why honeybees would visit *Helicrysum* spp. plants; theories include rest from the pressure of the wind when visiting the dunal habitat or the gathering of resins for different purposes like propolis production^75^. Another theory is that the plants Volatiles Organic Compounds (VOCs) move as particulate due to the sea breeze surrounding the apiary similarly to pesticides^76^. When the volatile profiles of foreshore honey and different *Helicrysum* species from the same area were compared^77^, only five volatile organic compounds (VOCs) were found in both. These were hexanal, 3-hexen-1-ol, hexanal, octanal, and α-terpineol. Although, in more recent research, one of the most odour-active constituents of *Helichrysum italicum* subsp. *italicum* perceived was the monoterpenic alcool nerol^78^, and nerolic acid was one of the compounds found in both our samples. However, in several cases already investigated the nectar VOCs profile does not match with the one of honey, and compounds coming directly from the flower but not present in the nectar were already found comparing honey and plants VOCs^79^. None of the plants of the dunal system was found as principal or secondary pollen (and then principal or secondary nectar source), neither *Helycrysum* spp., but all of them contributed to honey features. The designation of “*Helycrysum* honey” on labels or the attribution of this honey features exclusively to the spread presence of *Helycrysum* spp. are not correct since attributing features to a main nectar source is possible just for monofloral honeys.

In our work, honey samples produced over two years were quite similar (Fig. 2). The main difference was attributable to the higher presence of acetic acid, mainly due to the fermentation processes and conservation and not attributable to the floristic origin (Fig. 2, Table S2). Some of the main compounds were propanoic acid, isobutyric acid and butyric acid, described as “pungent, acidic, rancid, cheesy, vinegar” while 2-methylbutyric acid is usually associated to a “fruity, sour, cheesy in dilution; acidic, sweaty, berry-like” scent. The floral note of honey, remembering an herbal tea, is probably due to the presence of log chained carboxylic acids and again to umbelliferone, that is one of the main compounds found in *Matricaria chamomilla*^80^ and could be present in some other *Compositae* species of the dune system, as *Anthemis maritima* L. (seashore chamomile). Some other interesting organic compounds were Nicotinic acid and its analogues (2-hydroxynicotinic acid and 2-hydroxyquinoline), which are involved in the biosynthesis of alkaloids, which effects on honeybees and other pollinator behaviours are being deeply investigates^81–85^.

We can reasonably conclude that the organoleptic and aroma profile of honey produced in Migliarino-San Rossore-Massaciuccoli Regional Park is created not only by *Helichrysum* spp., but by all the flora composing the ecosystem of the area, included the allochthonous and ruderal, that are even the main floral component, and that the analytical support was important to well-describe this kind of honey, integrating the information of beekeepers and land managers, as MiPAF. Honeybees were already assessed as bioindicators of changing environments, as agricultural landscapes^86^ or even pollution and climate change^87^, and bee products as honey could assume the same role in the future. As for other kinds of bee products of fragile habitats, honey produced in an exceptional territory becomes the picture of the vegetation of unique and fragile ecosystems and the environmental and social changes they are facing, as abandonment^5^ or urbanization and allochthonous invasions, as in the case here described.

## Conclusions

Our research pointed out the importance of multifaceted analytical approaches for the characterization and better definition of traditional agri-food products as honey. Herein, honey from Migliarino-San Rossore-Massaciuccoli Park showed interesting features and was a good representation of the vegetation of this area, also for what concerns the threats to the integrity of the ecosystems, as degradation and spread of allochthonous and ruderal plants. The phytochemical composition was as well very interesting, abundant in variety and quantity in phenolic compounds. Some interesting compounds were long chain hydroxyacids characteristic of royal jelly and coumarine derivatives as 4-hydroxycoumarine and 7-hydroxycoumarine, that, together with VOCs constituent the aroma profile, are responsible of the particularity of the honey produced in this area of naturalistic, economic, and touristic importance. Given the strong linkage with the floral origin, honey is a representation of the changes happening in different and fragile habitats, as the Italian sand dunes system. This research testifies also the difficult of describing complex food matrixes as bee products, that are strongly affected by a changing environment. It is also advisable a more consistent interaction between beekeepers and a technical and analytical support, for the correct attribution of the labels to the products, the base for their valorisation.

### Supplementary Information

Supplementary Tables.

## Data Availability

The authors confirm that the data supporting the findings of this study are available within the article [and/or] its supplementary materials.
