# Supplementary material for: Formal analyses are fundamental for the definition of honey, a product representing specific territories and their changes: the case of North Tyrrhenian dunes (Italy)
Source: Sci Rep. 2023 Oct 16;13:17542. doi: 10.1038/s41598-023-44769-1 (PMC10579322; doi:10.1038/s41598-023-44769-1)
Supplement: Supplementary file 1 — Supplementary Tables. [file 41598_2023_44769_MOESM1_ESM.pdf]

Table S1. Metabolomic identification of secondary metabolites (Rt, retention time) detected in the two honey samples analyzed (2021 and 2022); values are in ppm.

Compounds	Formula	m/z	RT	2021	2022	p-value	Sign. Code
Carboxylic acids							
1-(Carboxymethyl)cyclohexanecarboxylic acid	$C_9H_{14}O_4$	185.08	10.44	0.70 ± 0.04	0.80 ± 0.06	0.0977	ns
1,2,3-cyclopropanetricarboxylic acid	$C_6H_6O_6$	173.01	2.75	1.87 ± 0.16	2.08 ± 0.06	0.0978	ns
1,2,4-Benzenetricarboxylic acid	$C_9H_6O_6$	209.01	6.39	2.21 ± 0.21	1.02 ± 0.06	0.0007	**
10-HDA	$C_{10}H_{18}O_3$	185.12	11.53	0.89 ± 0.04	0.83 ± 0.05	0.1588	ns
12-Hydroxydodecanoic acid	$C_{12}H_{24}O_3$	199.17	13.7	3.46 ± 0.28	3.30 ± 0.18	0.4653	ns
13-HODE	$C_{18}H_{32}O_3$	295.23	15.68	3.77 ± 0.46	2.62 ± 0.36	0.0269	*
13-Isogrindelic acid	$C_{20}H_{32}O_3$	319.23	15.44	7.01 ± 1.67	6.06 ± 0.86	0.4268	ns
14(Z)-Eicosenoic acid	$C_{20}H_{38}O_2$	309.28	18.15	9.11 ± 0.74	3.60 ± 0.25	0.0003	**
16-Hydroxyhexadecanoic acid	$C_{16}H_{32}O_3$	271.23	15.66	13.69 ± 2.31	8.81 ± 0.31	0.0222	*
2,2-Dimethylsuccinic acid	$C_6H_{10}O_4$	145.05	7.84	0.57 ± 0.03	0.63 ± 0.04	0.0739	ns
2,3,4,9-Tetrahydro-1H-β-carboline-3-carboxylic acid	$C_{12}H_{12}N_2O_2$	215.08	7.47	0.57 ± 0.02	0.49 ±0.02	0.0109	*
2-[(2S,4aR,8aS)-2-Hydroxy-4a-methyl-8-methylenedecahydro-2-naphthalenyl]acrylic acid	$C_{15}H_{22}O_3$	249.15	16.23	5.34 ± 4.08	2.31 ± 0.26	0.2690	ns
2-Furoic acid	$C_5H_4O_3$	113.02	12.18	9.31 ± 1.30	7.68 ± 0.22	0.0991	ns
2-Hydroxynicotinic acid	$C_6H_5NO_3$	138.02	7.37	3.14 ± 0.10	2.88 ± 0.14	0.0579	ns
2-Hydroxyvaleric acid	$C_5H_{10}O_3$	101.06	5.95	3.99 ± 0.09	4.95 ± 0.09	0.0002	**
2-Isopropylmalic acid	$C_7H_{12}O_5$	175.06	7.8	18.83 ± 0.89	18.89 ± 0.10	0.9037	ns
2-Methyl-3-hydroxybutyric acid	$C_5H_{10}O_3$	117.06	5.77	7.01 ± 0.75	7.78 ± 0.25	0.1660	ns
2-Methylhippuric acid	$C_{10}H_{11}NO_3$	192.07	9.22	0.89 ± 0.03	1.36 ± 0.05	0.0002	**
2-Norbornaneacetic acid	$C_9H_{14}O_2$	153.09	11.43	1.29 ± 0.06	1.31 ± 0.02	0.7166	ns
3-(2-Hydroxy-3-methoxyphenyl)acrylic acid	$C_{10}H_{10}O_4$	195.07	9.6	3.34 ± 0.44	4.09 ± 0.37	0.0865	ns
3-(3,4-dimethoxyphenyl)prop-2-enoic acid	$C_{11}H_{12}O_4$	209.08	10.85	13.33 ± 1.17	15.80 ± 0.44	0.0269	*
3,3-Dimethylglutaric acid	$C_7H_{12}O_4$	159.07	9.15	0.66 ± 0.13	0.73 ± 0.05	0.4198	ns
3,5-Dihydroxybenzoic acid	$C_7H_6O_4$	153.02	7.3	0.48 ± 0.02	0.44 ± 0.04	0.2330	ns
3-Hydroxy myristic acid	$C_{14}H_{28}O_3$	243.2	15.81	0.57 ± 0.24	0.30 ± 0.06	0.1428	ns
3-Methylglutaric acid	$C_6H_{10}O_4$	145.05	7.62	0.78 ± 0.06	1.06 ± 0.07	0.0073	**
3-Methylpimelic acid	$C_8H_{14}O_4$	173.08	5.81	0.65 ± 0.04	0.71 ± 0.02	0.0816	ns
4-{[-3-(3,4-dihydroxyphenyl)prop-2-enoyl]oxy}-1,3,5-trihydroxycyclohexane-1-carboxylic acid	$C_{16}H_{18}O_9$	355.1	7.83	1.30 ± 0.02	1.61 ± 0.05	0.0005	**
4-Hydroxy-4-{4-hydroxy-2-[(1E)-6-hydroxy-1-hepten-1-yl]cyclopentyl}-2-butenoic acid	$C_{16}H_{26}O_5$	297.17	13.5	3.45 ± 0.06	4.26 ± 0.08	0.0002	**

4-Hydroxybenzoic acid	$C_7H_6O_3$	137.02	10.55	13.79 ± 0.55	13.38 ± 0.25	0.3106	ns
4-Phenylbutyric acid	$C_{10}H_{12}O_2$	165.09	11.64	24.04 ± 0.80	20.93 ± 0.18	0.0028	**
4-Toluic acid	$C_8H_8O_2$	135.04	8.18	2.15 ± 0.06	2.44 ± 0.07	0.0053	**
5-Hydroxyindole-3-acetic acid	$C_{10}H_9NO_3$	190.05	8.95	11.51 ± 0.67	11.57 ± 0.26	0.8859	ns
6-Quinolinecarboxylic acid	$C_{10}H_7NO_2$	174.05	4.34	2.42 ± 0.11	1.64 ± 0.08	0.0006	**
9-HpODE	$C_{18}H_{32}O_4$	311.22	14.75	1.04 ± 0.01	1.25 ± 0.16	0.0787	ns
Abscisic acid	$C_{15}H_{20}O_4$	247.13	11.43	23.08 ± 0.72	24.11 ± 0.34	0.0884	ns
Arachidic acid	$C_{20}H_{40}O_2$	311.3	18.9	1.77 ± 1.02	0.54 ± 0.08	0.1037	ns
Caffeic acid	$C_9H_8O_4$	179.03	8.18	68.61 ± 1.26	80.72 ± 1.09	0.0002	**
Camphanic acid	$C_{10}H_{14}O_4$	197.08	10.92	3.11 ± 0.13	3.60 ± 0.18	0.0191	*
Citric acid	$C_6H_8O_7$	191.02	2.21	227.78 ± 4.86	213.84 ± 3.98	0.0184	*
Crotonic acid	$C_4H_6O_2$	87.04	0.63	2.95 ± 0.31	2.86 ± 0.41	0.7827	ns
Cyclohexanecarboxylic acid	$C_7H_{12}O_2$	127.08	9.17	0.94 ± 0.05	1.06 ± 0.05	0.0346	*
D-(-)-Quinic acid	$C_7H_{12}O_6$	193.07	1.48	2.32 ± 0.10	0.43 ± 0.03	< 0,0001	***
Docosanoic Acid	$C_{22}H_{44}O_2$	339.33	20.07	1.29 ± 0.06	0.66 ± 0.02	< 0,0001	***
Dodecanedioic acid	$C_{12}H_{22}O_4$	229.14	13.57	6.46 ± 0.39	7.21 ± 0.42	0.0860	ns
Ethylmalonic acid	$C_5H_8O_4$	131.03	5.65	5.68 ± 0.19	6.81 ± 0.35	0.0080	**
Fumaric acid	$C_4H_4O_4$	115	2.56	2.78 ± 0.49	2.52 ± 0.05	0.4154	ns
Gallic acid	$C_7H_6O_5$	169.01	3.88	2.81 ± 0.15	1.29 ± 0.06	< 0,0001	***
germacra-1(10),4,11(13)-trien-12-oic acid	$C_{15}H_{22}O_2$	233.15	14.98	13.87 ± 1.58	12.16 ± 0.30	0.1391	ns
Indole-3-acrylic acid	$C_{11}H_9NO_2$	188.07	7.8	0.53 ± 0.03	0.25 ± 0.02	0.0002	**
Lactic Acid	$C_3H_6O_3$	89.02	1.89	195.23 ± 30.70	180.29 ± 1.78	0.4474	ns
Levulinic acid	$C_5H_8O_3$	115.04	9.27	1.43 ± 0.05	1.76 ± 0.01	0.0003	**
Maleic acid	$C_4H_4O_4$	117.02	14.46	5.31 ± 0.47	5.04 ± 0.11	0.3935	ns
Mesaconic acid	$C_5H_6O_4$	129.02	4.18	4.09 ± 0.57	4.61 ± 0.04	0.1923	ns
Methylmalonic acid	$C_4H_6O_4$	117.02	2.67	45.59 ± 1.78	41.00 ± 1.24	0.0213	*
Mevalonic acid	$C_6H_{12}O_4$	147.07	6	19.68 ± 1.32	24.90 ± 0.21	0.0025	**
Myristic acid	$C_{14}H_{28}O_2$	227.2	16.8	2.85 ± 0.74	2.22 ± 0.06	0.2125	ns
Nicotinic acid	$C_6H_5NO_2$	124.04	2.21	5.68 ± 0.38	9.43 ± 0.32	0.0002	**
Oleanolic acid	$C_{30}H_{48}O_3$	455.35	17.04	0.93 ± 0.01	0.25 ± 0.02	< 0,0001	***
Palmitic Acid	$C_{16}H_{32}O_2$	255.23	17.39	41.83 ± 2.59	44.24 ± 2.29	0.2941	ns
Palmitoleic acid	$C_{16}H_{30}O_2$	253.22	16.98	3.67 ± 0.46	2.90 ± 0.05	0.0454	*

Pentadecanoic acid	$C_{15}H_{30}O_2$	241.22	17.1	1.54 ± 0.33	1.33 ± 0.09	0.3310	ns
Protocatechuic acid	$C_7H_6O_4$	153.02	6.04	7.61 ± 0.15	5.04 ± 0.21	< 0,0001	***
Ricinoleic Acid	$C_{18}H_{34}O_3$	297.24	16.31	2.24 ± 0.83	1.21 ± 0.06	0.0997	ns
Salicylic acid	$C_7H_6O_3$	137.02	7.44	6.21 ± 0.64	5.74 ± 0.29	0.3101	ns
Suberic acid	$C_8H_{14}O_4$	173.08	9.82	40.46 ± 1.32	43.53 ± 1.00	0.0326	*
Flavonoids							
3-Methoxy-5,7,3',4'-tetrahydroxy-flavone	$C_{16}H_{12}O_7$	315.05	12.42	11.01 ± 0.75	9.58 ± 0.24	0.0349	*
Apigenin	$C_{15}H_{10}O_5$	269.04	13.78	51.41 ± 4.08	49.54 ± 1.59	0.5010	ns
Chrysin	$C_{15}H_{10}O_4$	253.05	15.39	7.00 ± 0.45	7.74 ± 1.44	0.4404	ns
Daidzein	$C_{15}H_{10}O_4$	253.05	13.75	56.84 ± 3.87	50.82 ± 1.09	0.0606	ns
Diosmetin	$C_{16}H_{12}O_6$	299.06	12.25	1.73 ± 0.14	0.82 ± 0.05	0.0004	**
Fisetin	$C_{15}H_{10}O_6$	285.04	11.86	1.97 ± 0.17	1.27 ± 0.05	0.0025	**
Formononetin	$C_{16}H_{12}O_4$	269.08	13.14	4.49 ± 0.06	3.96 ± 0.07	0.0005	**
Genistein	$C_{15}H_{10}O_5$	269.05	12.5	6.71 ± 0.66	6.11 ± 0.33	0.2307	ns
Glycitein	$C_{16}H_{12}O_5$	283.06	14.07	13.52 ± 1.28	12.41 ± 0.53	0.2383	ns
Hispidulin	$C_{16}H_{12}O_6$	299.06	12.71	7.01 ± 0.64	6.53 ± 0.34	0.3111	ns
Isorhamnetin	$C_{16}H_{12}O_7$	315.05	13.03	7.89 ± 1.28	7.04 ± 0.45	0.3381	ns
Kaempferol	$C_{15}H_{10}O_6$	285.04	11.29	0.08 ± 0.02	0.10 ± 0.01	0.1645	ns
licoricidin	$C_{26}H_{32}O_5$	423.22	16.93	1.02 ± 0.30	1.27 ± 0.33	0.3702	ns
Luteolin	$C_{15}H_{10}O_6$	285.04	12.24	7.85 ± 0.53	9.98 ± 0.39	0.0048	**
Naringenin	$C_{15}H_{12}O_5$	271.06	11.77	160.56 ± 6.87	190.61 ± 4.39	0.0031	**
olmelin	$C_{16}H_{12}O_5$	285.08	13.5	5.49 ± 0.36	5.50 ± 0.21	0.9542	ns
pinostrobin	$C_{16}H_{14}O_4$	271.1	14.72	2.68 ± 0.12	2.09 ± 0.07	0.0016	**
Quercetin	$C_{15}H_{10}O_7$	301.03	11.46	3.58 ± 0.30	3.29 ± 0.19	0.2157	ns
Strobopinin	$C_{16}H_{14}O_4$	269.08	13.24	0.45 ± 0.10	2.19 ± 0.22	0.0002	**
Tricin	$C_{17}H_{14}O_7$	329.07	13.44	19.67 ± 1.85	18.12 ± 0.80	0.2534	ns
Nitrogen compounds							
1,3-Dimethylpteridine-2,4-dione	$C_8H_8N_4O_2$	193.07	7.18	1.69 ± 0.06	0.67 ± 0.01	< 0,0001	***
1-Tetradecylamine	$C_{14}H_{31}N$	214.25	13.42	6.85 ± 0.44	6.15 ± 0.17	0.0635	ns
2-Hydroxyquinoline	C_9H_7NO	146.06	9.93	1.36 ± 0.06	1.40 ± 0.07	0.5046	ns
3-Methylquinolin-2-one	$C_{10}H_9NO$	160.08	5.38	1.74 ± 0.03	1.11 ± 0.13	0.0012	**
5-Methoxyindole	C_9H_9NO	148.08	7.06	0.39 ± 0.29	0.65 ± 0.03	0.1947	ns
6-Methoxyquinoline	$C_{10}H_9NO$	160.08	8.81	1.35 ± 0.04	1.47 ± 0.06	0.0537	ns

Betaine	$C_5H_{11}NO_2$	118.09	1.37	16.17 ± 0.26	16.44 ± 0.12	0.1757	ns
Caprolactam	$C_6H_{11}NO$	114.09	7.97	7.56 ± 2.59	5.49 ± 0.45	0.2445	ns
Choline	$C_5H_{13}NO$	104.11	1.28	17.02 ± 0.28	15.59 ± 0.31	0.0040	**
Di-4-coumaroylputrescine	$C_{22}H_{24}N_2O_4$	381.18	10.22	0.72 ± 0.06	0.30 ± 0.01	0.0002	**
Isoleucine	$C_6H_{13}NO_2$	132.1	2.63	19.33 ± 1.42	17.07 ± 0.25	0.0528	ns
Leucine	$C_6H_{13}NO_2$	132.1	2.8	16.94 ± 0.64	16.46 ± 0.09	0.2639	ns
Methyl indole-3-acetate	$C_{11}H_{11}NO_2$	190.09	6.95	10.31 ± 0.48	5.70 ± 0.08	< 0,0001	***
N-Acetyldopamine	$C_{10}H_{13}NO_3$	196.1	6.94	2.78 ± 0.19	2.27 ± 0.06	0.0121	*
N-Acetylglutamic acid	$C_7H_{11}NO_5$	188.06	2.64	1.01 ± 0.07	0.86 ± 0.02	0.0272	*
N-Acetylleucine	$C_8H_{15}NO_3$	172.1	8.96	3.40 ± 0.16	4.06 ± 0.15	0.0070	**
N-Acetylphenylalanine	$C_{11}H_{13}NO_3$	206.08	9.56	3.76 ± 0.18	4.87 ± 0.21	0.0022	**
N-Acetyltyramine	$C_{10}H_{13}NO_2$	180.1	7.91	0.74 ± 0.02	0.99 ± 0.05	0.0011	**
N-Acetyltyrosine	$C_{11}H_{13}NO_4$	222.08	7.23	1.82 ± 0.08	2.20 ± 0.14	0.0148	*
N-Acetylvaline	$C_7H_{13}NO_3$	160.1	7.18	1.72 ± 0.05	1.92 ± 0.07	0.0174	*
Phenylalanine	$C_9H_{11}NO_2$	166.09	5.21	175.27 ± 6.38	138.35 ± 2.99	0.0008	**
Prolylleucine	$C_{11}H_{20}N_2O_3$	229.15	1.94	10.07 ± 0.46	9.49 ± 0.35	0.1599	ns
Pyridoxine	$C_8H_{11}NO_3$	170.08	2.24	2.42 ± 0.05	2.21 ± 0.03	0.0032	**
Stachydrine	$C_7H_{13}NO_2$	144.1	1.5	20.33 ± 0.06	20.48 ± 2.07	0.9076	ns
Uracil	$C_4H_4N_2O_2$	113.03	2.91	5.22 ± 0.07	5.25 ± 0.06	0.5844	ns
Uridine	$C_9H_{12}N_2O_6$	243.06	2.92	5.55 ± 0.32	5.20 ± 0.15	0.1607	ns
Valine	$C_5H_{11}NO_2$	118.09	1.65	20.18 ± 1.67	19.33 ± 1.24	0.5160	ns
δ-Valerolactam	C_5H_9NO	100.08	6.39	2.61 ± 0.14	2.65 ± 0.08	0.6646	ns
Glycosides							
2-Methylbutyl beta-D-glucopyranoside	$C_{11}H_{22}O_6$	268.18	9.32	1.89 ± 0.12	1.55 ± 0.05	0.0128	*
2-Phenylethyl-glucopyranoside	$C_{14}H_{20}O_6$	307.12	9.31	0.36 ± 0.01	0.36 ± 0.03	0.7090	ns
Carbonyl compounds							
2,4,5-Trimethoxybenzaldehyde	$C_{10}H_{12}O_4$	197.08	10.69	0.99 ± 0.25	6.62 ± 0.08	< 0,0001	***
2,4,6-Trihydroxy-2-(4-hydroxybenzyl)-1-benzofuran-3(2H)-one	$C_{15}H_{12}O_6$	287.06	10.1	0.88 ± 0.03	1.15 ± 0.07	0.0031	**
3',11'-dihydroxy-1',2',5'-trimethyl-8'-oxaspiro[oxirane-2,12'-tricyclo[7.2.1.0]dodecan]-5'-en-4'-one	$C_{15}H_{20}O_5$	279.12	9.27	0.73 ± 0.06	0.77 ± 0.02	0.3270	ns
Benzophenone	$C_{13}H_{10}O$	183.08	13.65	617.25 ± 40.88	585.58 ± 2.82	0.2517	ns
Citral	$C_{10}H_{16}O$	153.13	12.4	4.61 ± 0.19	5.99 ± 0.03	0.0002	**
Coenzyme Q0	$C_9H_{10}O_4$	183.07	7.1	1.42 ± 0.05	1.90 ± 0.04	0.0002	**

curcumenone	$C_{15}H_{22}O_2$	233.15	0.56	0.29 ± 0.01	0.51 ± 0.57	0.5469	ns
gentisaldehyde	$C_7H_6O_3$	137.02	7.07	2.13 ± 0.07	2.50 ± 0.09	0.0055	**
Isobutyraldehyde	C_4H_8O	73.06	3.21	14.62 ± 1.29	15.53 ± 0.05	0.2880	ns
Kojic acid	$C_6H_6O_4$	143.03	5.65	2.33 ± 0.76	2.32 ± 0.10	0.9845	ns
Esters and lactones							
3,8,9-trihydroxy-10-propyl-3,4,5,8,9,10-hexahydro-2H-oxecin-2-one	$C_{12}H_{20}O_5$	243.12	10.53	1.98 ± 0.04	2.42 ± 0.11	0.0029	**
4-Hydroxycoumarin	$C_9H_6O_3$	163.04	13.8	214.25 ± 11.34	199.65 ± 2.13	0.0936	ns
6-Pentyl-2H-pyran-2-one	$C_{10}H_{14}O_2$	167.11	13.8	3.80 ± 0.50	3.53 ± 0.06	0.4130	ns
7-Hydroxycoumarine	$C_9H_6O_3$	163.04	15.39	507.08 ± 39.97	526.55 ± 22.96	0.5050	ns
Ascorbic acid	$C_6H_8O_6$	175.02	2.04	9.92 ± 0.98	9.52 ± 1.59	0.7279	ns
Mevalonolactone	$C_6H_{10}O_3$	131.07	4.27	3.48 ± 0.15	3.61 ± 0.07	0.2168	ns
Propyl gallate	$C_{10}H_{12}O_5$	211.06	6.36	31.21 ± 1.18	9.01 ± 0.35	< 0,0001	***
δ-Ribono-1,4-lactone	$C_5H_8O_5$	147.03	2.79	52.67 ± 2.04	63.29 ± 1.52	0.0019	**
Others							
1,2,3,4-Tetramethyl-1,3-cyclopentadiene	C_9H_{14}	123.12	11.95	1.06 ± 0.06	1.79 ± 0.01	< 0,0001	***
Galactal	$C_6H_{10}O_4$	145.05	6.96	0.97 ± 0.09	1.19 ± 0.05	0.0168	*
Pyrogallol	$C_6H_6O_3$	125.02	6.15	0.62 ± 0.01	0.87 ± 0.02	< 0,0001	***
Rishitin	$C_{14}H_{22}O_2$	221.15	15.47	6.80 ± 0.08	5.47 ± 2.78	0.4523	ns
Tetrahydrofuran	C_4H_8O	73.06	7.37	0.97 ± 0.05	0.80 ± 0.09	0.0504	ns
α-Pinene-2-oxide	$C_{10}H_{16}O$	153.13	7.79	0.85 ± 0.24	3.14 ± 0.41	0.0011	**

Table S2. Identification of volatile organic compounds (VOCs) detected in in the two honey samples analyzed (2021 and 2022). Rt=retention time; values are in ppm.

Compounds	CAS	RT	2021	2022	p-value	Signific. Code
Alcohols						
Ethanol	64-17-5	3.77	4.01 ± 0.14	1.57 ± 0.06	<0.0001	***
2-butanol	78-92-2	6.5	1.55 ± 0.15	0.75 ± 0.02	0.0008	**
1-propanol	71-23-8	7.05	5.39 ± 1.45	3.27 ± 0.06	0.0646	ns
2-methylbut-3-en-2-ol	115-18-4	7.25	4.40 ± 1.22	2.35 ± 0.22	0.0457	*
Isobutanol	78-83-1	10.14	0.55 ± 0.02	0.42 ± 0.03	0.0032	**
2-pentanol	6032-29-7	11.51	0.13 ± 0.01	0.11 ± 0.01	0.0818	ns
1-butanol	71-36-3	12.57	0.92 ± 0.06	0.70 ± 0.01	0.0030	**
2-hexanol	626-93-7	13.72	0.06 ± 0.01	0.07 ± 0.01	0.2197	ns
3-methyl-1-butanol	123-51-3	15.34	12.83 ± 1.06	8.62 ± 0.36	0.0029	**
3-methyl-3-buten-1-ol	763-32-6	16.71	4.73 ± 0.04	4.54 ± 0.26	0.2825	ns
2-methyl-but-2-ene-1-ol	4675-87-0	19.21	2.10 ± 0.22	0.68 ± 0.22	0.0014	**
3-pentanol	584-02-1	19.36	0.39 ± 0.02	0.71 ± 0.07	0.0013	**
1-hexanol	111-27-3	20.34	5.20 ± 1.00	4.31 ± 0.54	0.2455	ns
3-hexen-1-ol	544-12-7	21.07	10.41 ± 2.32	5.57 ± 0.63	0.0251	*
p-cymen-7-ol	536-60-7	30.41	0.80 ± 0.02	0.48 ± 0.03	<0.0001	***
2-phenylethanol	60-12-8	31.6	9.70 ± 2.11	3.58 ± 0.26	0.0075	**
Aldehydes						
2-methylbutanal	96-17-3	3.33	2.13 ± 0.48	1.74 ± 0.17	0.2629	ns
3-methylbutanal	590-86-3	3.4	0.96 ± 0.06	1.05 ± 0.11	0.2782	ns
Hexanal	66-25-1	8.94	1.00 ± 0.08	0.55 ± 0.11	0.0048	**
2-methyl-2-butenal	1115-11-3	9.26	4.49 ± 0.19	3.71 ± 0.02	0.0020	**
Heptanal	111-71-7	14.02	0.08 ± 0.01	0.06 ± 0.02	0.0894	ns
3-methyl-2-butenal	107-86-8	14.45	1.97 ± 0.57	1.84 ± 0.28	0.7419	ns
2-hexenal	645-62-5	15.19	0.93 ± 0.13	0.90 ± 0.19	0.8301	ns
Octanal	124-13-0	17.95	0.22 ± 0.02	1.00 ± 0.07	<0.0001	***
Furfural	98-01-1	22.57	8.16 ± 1.00	4.45 ± 0.27	0.0035	**
Benzaldehyde	100-52-7	23.84	10.84 ± 0.40	11.71 ± 0.17	0.0250	*
2-furanacetaldehyde	15022-16-9	25.27	0.59 ± 0.05	1.76 ± 0.07	<0.0001	***
Phenylacetaldehyde	122-78-1	26.37	1.09 ± 0.32	0.76 ± 0.01	0.1509	ns
4,5-dimethylfurfural	52480-43-0	32.16	2.67 ± 0.61	3.12 ± 0.23	0.2970	ns
Cinnamaldehyde	14371-10-9	32.81	0.71 ± 0.17	0.57 ± 0.04	0.2483	ns
3,4-dimethoxy-benzaldehyde	120-14-9	37.07	0.10 ± 0.03	0.70 ± 0.02	<0.0001	***
Ketones						
Acetone	67-64-1	2.25	2.84 ± 0.35	1.38 ± 0.15	0.0026	**
2-butanone	78-93-3	3.13	13.78 ± 0.48	11.05 ± 0.86	0.0087	**
2,3-butanedione	431-03-8	4.82	1.08 ± 0.14	0.92 ± 0.05	0.1352	ns
3-penten-2-one	3102-33-8	5.04	0.77 ± 0.11	0.80 ± 0.02	0.7421	ns
2,3-pentanedione	600-14-6	8.14	0.14 ± 0.01	0.19 ± 0.01	0.0032	**
2-heptanone	110-43-0	13.86	0.10 ± 0.03	0.36 ± 0.46	0.3868	ns

cyclohexanone	108-94-1	17.46	0.26 ± 0.05	0.09 ± 0.03	0.0074	**
3-hydroxy-2-butanone	51555-24-9	17.6	1.07 ± 0.01	0.95 ± 0.24	0.4411	ns
Hydroxyacetone	116-09-6	18.05	2.28 ± 0.21	2.30 ± 0.38	0.9555	ns
2-furyl methyl ketone	1192-62-7	23.48	0.56 ± 0.05	0.64 ± 0.08	0.2049	ns
Ketoisophorone	1125-21-9	27.38	1.65 ± 0.24	1.45 ± 0.03	0.2298	ns
Carboxylic acids						
Acetic acid	64-19-7	21.73	333.11 ± 14.10	292.41 ± 4.58	0.0089	**
Formic acid	64-18-6	23.29	3.04 ± 0.12	2.19 ± 0.08	0.0006	**
Propanoic acid	79-09-4	24.13	26.06 ± 1.60	26.08 ± 0.47	0.9855	ns
Isobutyric acid	79-31-2	24.88	9.76 ± 1.37	9.95 ± 0.22	0.8214	ns
Butyric acid	107-92-6	26.13	4.95 ± 0.82	4.86 ± 0.42	0.8772	ns
2-methylbutanoic acid	116-53-0	26.99	32.33 ± 2.09	28.41 ± 0.52	0.0346	*
Pentanoic acid	109-52-4	28.3	0.60 ± 0.01	0.55 ± 0.08	0.3083	ns
3-methyl-2-butenoic acid	541-47-9	29.36	1.92 ± 0.28	1.47 ± 0.09	0.0576	ns
4-methylpentanoic acid	646-07-1	29.53	0.30 ± 0.03	0.11 ± 0.01	0.0004	**
Caproic acid	142-62-1	30.3	6.53 ± 0.04	5.90 ± 0.44	0.0691	ns
Heptanoic acid	111-14-8	31.94	2.56 ± 0.28	1.87 ± 0.44	0.0835	ns
3-hexenoic acid	1577-18-0	31.97	2.25 ± 0.35	2.09 ± 0.31	0.5958	ns
2-hexenoic acid	1191-04-4	32.06	2.41 ± 0.30	1.27 ± 0.05	0.0029	**
Octanoic acid	124-07-2	33.03	3.68 ± 1.46	1.02 ± 0.69	0.0461	*
Nonanoic acid	112-05-0	34.1	5.37 ± 2.30	3.14 ± 0.83	0.1894	ns
Benzoic acid	65-85-0	37.79	2.08 ± 0.39	1.97 ± 0.06	0.6523	ns
Esters						
Methyl acetate	79-20-9	2.37	0.41 ± 0.05	0.59 ± 0.07	0.0240	*
Ethyl acetate	141-78-6	3	1.45 ± 0.03	0.43 ± 0.05	<0.0001	***
Propyl acetate	109-60-4	4.73	0.88 ± 0.05	0.47 ± 0.05	0.0005	**
2-methylbutyl acetate	624-41-9	11.19	0.19 ± 0.02	0.10 ± 0.01	0.0020	**
Isoamyl acetate	123-92-2	11.27	0.36 ± 0.06	0.16 ± 0.02	0.0040	**
4-pentenyl acetate	1576-85-8	14.72	0.11 ± 0.02	0.13 ± 0.02	0.3512	ns
cis-3-hexenyl acetate	3681-71-8	18.93	0.06 ± 0.01	0.10 ± 0.01	0.0060	**
Ethyl lactate	97-64-3	19.65	0.13 ± 0.01	0.07 ± 0.01	0.0062	**
γ-valerolactone	108-29-2	25.58	2.94 ± 0.59	2.18 ± 0.05	0.0896	ns
4-methyl-4-vinylbutyrolactone	1073-11-6	26.83	1.27 ± 0.32	0.85 ± 0.09	0.0924	ns
(*D*)-pantolactone	599-04-2	32.7	1.30 ± 0.33	1.59 ± 0.09	0.2142	ns
(*R*)-(-)-massoilactone	51154-96-2	34.84	0.34 ± 0.06	1.25 ± 0.10	0.0002	***
Terpenes						
Citral	5392-40-5	15.47	2.89 ± 0.33	3.24 ± 0.46	0.3311	ns
Herboxide	13679-86-2	16.4	0.12 ± 0.02	0.21 ± 0.01	0.0049	**
trans-linalool oxide	34995-77-2	22.83	1.35 ± 0.20	0.96 ± 0.03	0.0296	*
Linalool	78-70-6	24.67	3.36 ± 0.95	4.71 ± 0.15	0.0714	ns
Hotrienol	20053-88-7	25.94	7.73 ± 1.12	6.74 ± 0.18	0.2019	ns
α-terpineol	98-55-5	27.61	0.84 ± 0.13	0.64 ± 0.06	0.0817	ns
Nerolic acid	4613-38-1	35.97	0.64 ± 0.11	0.32 ± 0.02	0.0076	**
Others						
Octane	111-65-9	2.18	0.65 ± 0.10	0.70 ± 0.12	0.6492	ns
2-methoxy-2-methylpropane	1634-04-4	1.7	1.42 ± 0.03	1.68 ± 0.04	0.0011	**
Dimethyl sulfide	75-18-3	1.9	5.43 ± 0.56	2.68 ± 0.16	0.0012	**
Dimethyldisulfide	624-92-0	8.28	0.67 ± 0.02	0.19 ± 0.04	<0.0001	***

2,5-dimethylpyrazine	123-32-0	18.81	0.07 ± 0.01	0.33 ± 0.06	0.0021	**
2,6-dimethylpyrazine	108-50-9	19.02	0.09 ± 0.02	0.15 ± 0.02	0.0215	*
2-ethyl-3-methylmaleic anhydride	3552-33-8	28.2	1.30 ± 0.57	1.05 ± 0.19	0.5131	ns
2-methoxy-4-vinylphenol	7786-61-0	34.41	0.82 ± 0.13	0.59 ± 0.06	0.0489	*
